# Environment and health: how do we close the gap to prevent ill-health, poor well-being, and environmental degradation?

**DOI:** 10.14324/111.444/ucloe.000043

**Published:** 2022-09-15

**Authors:** Dan Osborn

**Affiliations:** 1 Chair of Human Ecology, Department of Earth Sciences, University College London, 5 Gower Place, London, WC1E 6BS, UK; 2 Editor-in-Chief, UCL Open: Environment

## The perspectives of frameworks and fora

The links between environment and health are well established but often neither the scientific nor the policy aspects of these fields are as integrated as they might be to draw on all the expertise available in these two large fields of study to deliver the best outcomes. This is particularly challenging given the wide range of proximal and distal factors that can affect health and wellbeing. How can the gaps between these two large fields of study be closed and the fora and frameworks created to enable these two disciplinary areas to collaborate on knowledge, theory, evidence-base, policy and practice?

There is some urgency about closing existing gaps and putting mechanisms in place to deal with any that arise because, as the world’s population grows and the environment comes under increasing pressure, health impacts on people, the health and social care systems, and even health shocks such as pandemics, may well increase in importance. In the past, humanity has tended to rely on technological developments to deal with its issues, but, as efforts under the UN Framework Convention on Climate Change (UNFCC) indicate, in fora such as COP26 (Conference of the Parties) and the Intergovernmental Panel on Climate Change (IPCC), we are short of time to deploy technological solutions alone.

**Figure 1:**
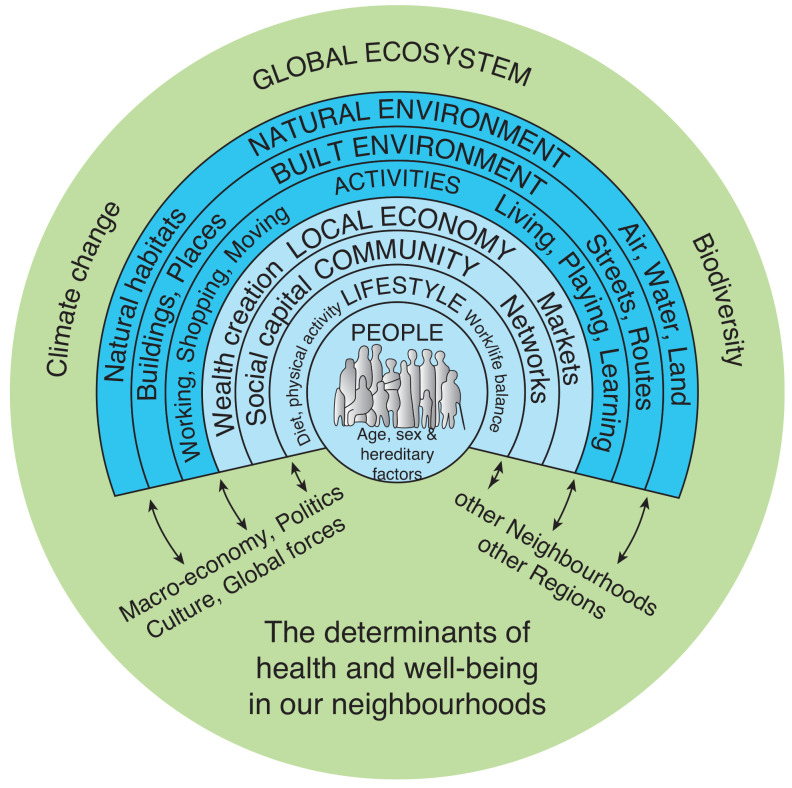
Rainbow model health map. Source: based on work in [1,2,3,31].

In 1991, Dahlgren and Whitehead [1] suggested a conceptual framework, which they have recently reviewed [2], setting out a view of the wider determinants of health, which has yet to be improved upon in its fundamentals. This includes, wrapped around all other factors, ‘general socio-economic, cultural and environmental conditions’. This basic ‘rainbow model’ has been developed over time, including by Barton et al. in their work on shaping neighbourhoods [3,4] to include more of the aspects that touch on both community factors and planetary health as in Fig. 1. Such conceptual models have been useful in policy and practice; for instance, the rainbow model health map (Fig. 1) being a foundation of the second UK climate change risk assessment evidence base dealing with people and the built environment [5] that informed the UK’s statutory 2017 climate change risk assessment.

There are numerous other frameworks around which the environment and health topics have been brigaded. Some of these address issues focused more on the internal environment of an organism (e.g., [6]) than the organism’s external environment. Interestingly, even when words, such as ecology, having their most usual scientific usage grounded in the open, external, environment are used as part of ecological models of public health (e.g., [7,8]), these models can refer little, if at all, to the environmental factors that might influence health. They may, in some senses, include environmental factors such as the unexpected changes to people’s living and working conditions that were probably important during the pandemic as they sometimes consider effects at community levels. Not always do such models deal with factors linked to decisions and choices made by governments and individuals, many of which are related to the open, external, environment (e.g., access to and use of green space which can be a boon to health and wellbeing and help prevent disease; for example, [9,10]).

Despite these gaps and conceptual uncertainties in frameworks and conceptual models, a great deal of work is already in hand on environment and health topics or in the links between the evidence base and policies affecting both areas. For example, after the initial University College London (UCL)–Lancet Commission report [11] there has been work in various fora on climate change and health, including within the IPCC and in national climate change risk assessments (e.g., [12]). Publications covering many aspects of health research and practice can now easily be found in the literature (e.g., [13–17]) and it will be interesting to see how studies funded through the Wellcome Trust’s new £75m investment in climate and health deal with the environment and health interface.

Thus, the environment–health interface is considerable, and its growing influence is reflected in the establishment of several fora such as those dealing with the One Health and planetary health concepts. These efforts are making it possible to quantify the importance of the interaction. For example, the World Health Organization (WHO) runs reporting systems that have enabled it to attribute 20% of deaths in the European Region to environmental factors^1^ of which air pollution may be the most readily quantifiable and identifiable. This figure rises to about 25% in the 2022 update [18]: a Compendium of guidance in health and environment interactions prepared in support of both the WHO Global Strategy on Health, Environment and Climate Change and the 2030 Agenda for Sustainable Development. The overarching purpose of the WHO Compendium [18] is to help countries to develop health protection and improvement policies and to address environmental risks through a shift towards primary preventative actions and the promotion of healthy choices. The environmental factors it covers include water (including sanitation and good hygiene), air pollution, noise, chemical and radiation exposure, housing, recreational risks (such as drownings), risks linked to the way land is used (including that from buildings), and others involving the workplace or communities and climate change. But the WHO Compendium has a number of different ways of dealing with the environment and this illustrates the difficulty of settling on what ‘the environment’, viewed from a human health perspective, actually means and what elements of risk to people’s health and wellbeing fall within the scope of such governmental documentation or, indeed, related fields of research. Despite the apparent importance of the environment in human health and mortality, the WHO’s headline data on Global Health Estimates^2^ seem not to address the environment explicitly, being concerned with identifying medical conditions that are the leading cause of death and not the wider social, economic or environmental factors that lead or contribute to these conditions affecting so many people worldwide. However, in the detail, a number of environmental factors and classifications are apparent.

All this suggests that what is happening at the environment–health interface is a case of proximal causes getting more attention than distal ones, especially if the proximal causes are easier to quantify, create hypotheses around or design interventions for. And, of course, such proximal studies may also be easier to fund as they more readily meet criteria for grants and awards – or even academic publication. This general argument is probably as true for studies that are mainly environmental as it is for studies with a health focus. Indeed, once knowledge reaches a certain level and a certain type then it is possible to argue that both health and environment can use similarly rigorous approaches to manage problems, for example, through systematic review processes (for examples in both areas see [19,20]). But such work does focus on the proximal and can lean towards technological solutions that can only be part of the solution.

Overall, navigating the environment and health interface is complex for all those concerned with understanding and/or lessening the burden of disease arising from environmental sources that varies in importance in different parts of the world. These are issues on the agenda of contributors to the WHO’s Global Health Observatory^3^ but how much the environmental aspects can be given attention must in part depend on the availability of resources that, under current paradigms, have to be devoted primarily to the pressing immediate medical needs of national and local populations.

## Covid-19: a spur to clarification of interlinkages between environment and health

In early 2020, environment and health interactions were forced on the attention of every government in the world once WHO expert groups considered that Covid-19 impacts and infections justified use of the term pandemic. Governments were forced to act quickly on an issue arising from what proximally was a health and medical emergency. Variants of the original virus – a zoonosis that jumped from wildlife to humans [21] – continue to cause illness and death to the present day and will continue to do so for some time despite the advent of vaccines and treatments. Perhaps the last time governments across the globe acted quickly on an environment issue was to put the Montreal Protocol in place – again this was to prevent thinning of the stratospheric ozone layer by CFC refridgerants and prevent skin cancer rates climbing even more sharply. Perhaps the lesson here is that health and environment issues taken together are what most easily enables global government action – after all, we all have an interest in the common future [22].

An earlier editorial [23] indicated this journal’s interest in what would be learnt about the interactions between health and the environment from the Covid-19 pandemic. Several of the articles in the special series linked to that editorial deal with the way the environment in which people live and work was changing as a consequence of the pandemic. Perhaps picking up from that editorial’s themes on how changes to people’s environment can affect their mental health [24], has now set out in this journal the rationale, within the UCL–Penn global study on Covid-19, for examining the ways the pandemic affected people’s mental health in their living environments changed and made more challenging by restrictions linked to Covid-19 control measures. Readers will be able to see the individual papers with their clear health focus placed in the broader context of both the series editorial and the views of invited discussants recording the policy relevance and study implications about the lessons learned, some of whom are experts from outside the academic community^4^.

Taking account of the environmental factors involved in such studies is probably more than can be tackled within any one data analysis simply because the environment can be defined so broadly – arguably as the totality of the physical, biological and chemical systems (some created by people) within which human societies operate. This means it is very likely, for the present, that the environment will tend to be treated as an implicit aspect of such studies.

Furthermore, responses to a crisis in public health mean it is the immediate impacts that need attention in the short-to-medium term and it is only in the medium-to-long term that work on the preventative and improvement measures sought by the WHO in its Compendium might come to the fore. Care will be needed to avoid the human tendency to relax once a crisis has passed and life seems to be more manageable because a degree of normality has been restored tends to work against any wish to invest in the kinds of improvements and preventative measures the WHO seeks as part of its Compendium of health measures.

Given the complexity of the issues involved in integrating environment and health that could impede progress, there could be advantages in making environmental factors more explicit than they sometimes are in studies of health, perhaps co-creating work with communities that will have detailed knowledge of their local environment as community involvement can play such a major role in improving health and resolving and identifying health inequalities (e.g. [25,26])^5^. And, ‘the environment’ itself needs some clarification as to what the term covers – for example, does the environment extend to or include Outer Space? In this journal’s experience thus far, it clearly involves more than what some would call ‘Nature’ or the ‘Natural World’.

Delaying dealing with distal, or the ultimate, causes of low or unequal health and wellbeing which would often involve preventative interventions and different choices by governments and individuals, only means that the costs of dealing with the proximal causes will rise and make equity that much more difficult to achieve and sustain. It seems likely that any hesitancy in taking action will hold back human development and mean crisis management remains too much of the norm. This, however, is nothing new. The point has been made time and again that there are delays in the response of any complex system to change and that the earlier action is taken to correct imbalances, or to avoid approaching environmental or social limits or tipping points [25,27,28], the more likely it is that we will have a healthy planet necessary to support a healthy population with high wellbeing.

It is my hope that the special series the journal is running presently on water [29], community responses to climate change [30], mould in the built environment^6^ and that on Covid-19 [23] and mental health in the environments in which we live [24] will all help provide the knowledge and evidence needed to spur action and reduce the delays being seen in the response of governments and society to the real challenges that people face all around the world.

## Notes

1. See: https://www.euro.who.int/__data/assets/pdf_file/0006/185217/Social-and-environmental-determinants-Fact-Sheet.pdf and https://www.who.int/europe/health-topics/environmental-health#tab=tab_1

2. See: https://www.who.int/data/global-health-estimates

3. See: https://www.who.int/data/gho/data/themes/public-healthand-environment with mapped information at https://www.who.int/data/gho/data/indicators/indicator-details/GHO/disability-adjusted-life-years-(dalys)-attributable-to-the-environment

4. See: https://www.doi.org/10.14293/S2199-1006.1.SOR-EARTH.CLIEN0H.v1

5. See https://ucl-about.scienceopen.com/covid-19-and-mental-health

6. See: https://doi.org/10.14293/S2199-1006.1.SOR-UNCAT.CLEMRZS.v1
